# Combined alteration of lamin and nuclear morphology influences the localization of the tumor-associated factor AKTIP

**DOI:** 10.1186/s13046-022-02480-5

**Published:** 2022-09-13

**Authors:** Mattia La Torre, Chiara Merigliano, Klizia Maccaroni, Alexandre Chojnowski, Wah Ing Goh, Maria Giubettini, Fiammetta Vernì, Cristina Capanni, Daniela Rhodes, Graham Wright, Brian Burke, Silvia Soddu, Romina Burla, Isabella Saggio

**Affiliations:** 1 Department Biology and Biotechnology, Sapienza University, Rome, Italy; 2 Molecular and Computational Biology Department, University of Southern California, Los Angeles, CA USA; 3 Nuclear Dynamics and Architecture, A*STAR Skin Research Labs (ASLR), Agency for Science, Technology and Research, A*STAR, Singapore, 639798 Singapore; 4 A*STAR Microscopy Platform, Research Support Centre, Agency for Science, Technology and Research, Singapore, 639798 Singapore; 5 CrestOptics S.P.A., Rome, Italy; 6 CNR Institute of Molecular Genetics “Luigi Luca Cavalli-Sforza”, Unit of Bologna, Bologna, 40136 Italy; 7 IRCCS Istituto Ortopedico Rizzoli, 40136 Bologna, Italy; 8 NTU Institute of Structural Biology, Nanyang Technological University, Singapore, Singapore; 9 MRC Laboratory of Molecular Biology, Cambridge Biomedical Campus, Cambridge, CB2 0QH UK; 10 Regina Elena National Cancer Institute IRCCS, Unit of Cellular Networks and Molecular Therapeutic Targets, Rome, Italy; 11 CNR Institute of Molecular Biology and Pathology, Rome, Italy

**Keywords:** Lamins, Nuclear morphology, Protein mislocalization, Risk biomarker, Tumor cells, Progeria mutations

## Abstract

**Background:**

Lamins, key nuclear lamina components, have been proposed as candidate risk biomarkers in different types of cancer but their accuracy is still debated. AKTIP is a telomeric protein with the property of being enriched at the nuclear lamina. AKTIP has similarity with the tumor susceptibility gene TSG101. AKTIP deficiency generates genome instability and, in p53^−/−^ mice, the reduction of the mouse counterpart of AKTIP induces the exacerbation of lymphomas. Here, we asked whether the distribution of AKTIP is altered in cancer cells and whether this is associated with alterations of lamins.

**Methods:**

We performed super-resolution imaging, quantification of lamin expression and nuclear morphology on HeLa, MCF7, and A549 tumor cells, and on non-transformed fibroblasts from healthy donor and HGPS (LMNA c.1824C > T p.Gly608Gly) and EDMD2 (LMNA c.775 T > G) patients. As proof of principle model combining a defined lamin alteration with a tumor cell setting, we produced HeLa cells exogenously expressing the HGPS lamin mutant progerin that alters nuclear morphology.

**Results:**

In HeLa cells, AKTIP locates at less than 0.5 µm from the nuclear rim and co-localizes with lamin A/C. As compared to HeLa, there is a reduced co-localization of AKTIP with lamin A/C in both MCF7 and A549. Additionally, MCF7 display lower amounts of AKTIP at the rim. The analyses in non-transformed fibroblasts show that AKTIP mislocalizes in HGPS cells but not in EDMD2. The integrated analysis of lamin expression, nuclear morphology, and AKTIP topology shows that positioning of AKTIP is influenced not only by lamin expression, but also by nuclear morphology. This conclusion is validated by progerin-expressing HeLa cells in which nuclei are morphologically altered and AKTIP is mislocalized.

**Conclusions:**

Our data show that the combined alteration of lamin and nuclear morphology influences the localization of the tumor-associated factor AKTIP. The results also point to the fact that lamin alterations per se are not predictive of AKTIP mislocalization, in both non-transformed and tumor cells. In more general terms, this study supports the thesis that a combined analytical approach should be preferred to predict lamin-associated changes in tumor cells. This paves the way of next translational evaluation to validate the use of this combined analytical approach as risk biomarker.

**Supplementary Information:**

The online version contains supplementary material available at 10.1186/s13046-022-02480-5.

## Background

AKTIP is a factor associated with cancer at multiple levels. Firstly, AKTIP and its mouse counterpart Ft1 are needed for telomere integrity. Indeed, depletion of AKTIP or Ft1 causes telomere fragility, DNA damage, and genome instability [1, 2]. Secondly, in vivo, Ft1 is associated with cancer invasiveness. Namely, in p53^−/−^ mice, depletion of Ft1 exacerbates lymphoma invasiveness to secondary sites [2]. Finally, AKTIP has sequence similarities with the protein TSG101, a tumor susceptibility gene [3]. We have shown that AKTIP, as TSG101, acts in association with the Endosomal Sorting Complex Required for Transport (ESCRT) complex [4], a membrane regulating machinery operating at the nuclear rim post-mitotically and at the midbody during cytokinesis [5].

The biology of AKTIP includes the unique property, as a telomere associated factor, to be stably enriched at the nuclear rim [1, 6]. In eukaryotic cells, the main constituents of the nuclear rim are the inner and outer membranes and the nuclear pores [7, 8]. Below the inner nuclear membrane there is the lamina. In mammalian cells, the lamina is constituted mainly of A- and B-type lamins. The *LMNA* gene encodes lamins A and C, whereas the *LMNB1* and *LMNB2* genes encode lamins B1 and B2, respectively. Imaging studies with high resolution microscopy have shown that lamins are organized into a meshwork structure [9, 10], which plays critical roles in the biology of the cell. The meshwork structure confers to the nucleus its mechanical properties, contributes to the organization of chromatin, and is associated with factors controlling nuclear functions [6, 11, 12].

Nuclear morphology and lamin alterations are present in malignant cells. Nuclear dysmorphisms have been proposed as diagnostic approach [13, 14], and the alteration of lamins has been reported in breast, ovarian, prostate, and colorectal carcinoma and in neuroblastoma [15]. Lamin mutations have also contributed to reveal the role of the ESCRT machinery to repair nuclear envelope ruptures occurring during cancer cell migration [16]. However, given the overall stratification of genetic and functional alterations in cancer, the molecular dissection of the impact of lamins on the disease is complex and multifaceted. Reduced levels of lamin A/C have been associated with poor prognosis, which has been mechanistically imputed to alterations of nuclear morphology in breast and ovarian cancers [17–19] and to increased cell motility in neuroblastoma [20]. On the other end, overexpression of lamin A/C has been observed in prostate and colorectal cancers where the alteration of lamins has been associated with an increased capability of cancer cells to invade surrounding tissues [21, 22].

A possible approach to correlate phenotypic traits to lamin mutations is the use of cells derived from laminopathic patients with identified mutations of the *LMNA* gene. Two examples are the Emery-Dreifuss Muscular Dystrophy (EDMD) and the Hutchinson Gilford Progeria Syndrome (HGPS). Autosomal EDMD has been linked to *LMNA* mutations as the LMNA c.775 T > G [23]. HGPS is due in most cases to heterozygous de novo C > T transition that exposes a cryptic splice site of the *LMNA* gene, generating the permanently farnesylated Δ50 variant of lamin A known as progerin [24]. The morphology of the nuclei is altered in both diseases, although differently, which reflects also on lamin-associated factors and on the clinical phenotypes [25–29].

Here, we asked whether the distribution of AKTIP is altered in cancer cells and whether this is associated with alterations of lamins. To this goal, we performed high resolution analyses of AKTIP in a set of cancer cell lines and in HGPS and EDMD lamin mutant cells. Performing the quantification of lamin expression and of nuclear morphology we observe that it is their combined alteration that influences the localization of the tumor-associated factor AKTIP. In general terms, these data support the thesis that a combined analytical approach should be used to predict lamin-associated intranuclear changes with potential tumor effects.

## Materials and methods

### Cells and viral vectors

HeLa (ATCC CCL-2), A549 (ATCC CCL-185) and MCF7 (ATCC HTB-22) and 293 T (ATCC CRL-11268) cells were cultured in DMEM (Invitrogen) supplemented with 10% FBS (Invitrogen). HGPS (HGADFN167, carrying LMNA c. 1824 C > T mutation) skin fibroblasts and their controls from relatives (HGMDFN090) were obtained from Progeria Research Foundation (PRF) Cells and Tissue Bank (Boston, MA, USA). Emery-Dreifuss muscular dystrophy (EDMD2, EDMD184, carrying LMNA c.775 T > G mutation) skin fibroblasts were obtained from the BioLaM biobank. The experimental protocol was approved by the local ethics committee (Rizzoli Orthopedic Institute Ethical Committee approval Prot. Gen. 0,018,250–2016) and followed EU regulations. Primary cells were used at low (between 7–9) passages and cells were cultured in DMEM (Invitrogen) supplemented with 20% FBS.

Second generation recombinant lentiviruses (LV) were produced and titrated as previously described [30] by co-transfection of 293 T cells with the vectors pCMV-dR8.74, pMD2.G (http://www.addgene.org) and a transfer vector. The transfer vector encoding progerin is pCDHblast MCSNard OST-LMNAd50 [31] and was provided by Addgene (Addgene plasmid 22,662); the transfer vector for control lentivector (LV-ctr) has been described previously [1]. The moi (molteplicity of infection measured as p24pg per cell) used for all experiments was 1. Transductions were performed in complete medium supplemented with 8ug/ml polybrene (Sigma). After viral addition, cells were centrifuged for 30 min at 1800 rpm at RT, incubated 1 h at 37 °C, and then transferred to fresh complete medium.

pCMV6-AC-AKTIP-GFP (Origene) and full length Flag-tagged prelamin A (LA-WT, pCI mammalian expression vector) [32] were transiently transfected into 293 T cells using the FuGene6 reagent (Promega). The transfection efficiency was over 75%. Cells were analyzed 24hs post transfection.

The population doubling was calculated with the formula Log(n_t_/n_0_) × 3.33, where n_0_ is the number of cells plated and n_t_ the number of cells after n days.

### Quantification of gene expression

One-week post-transduction, cells were lysed by addition of TRIzol reagent (Invitrogen) and RNA extracted according to the manufacturer’s instructions. After DNase treatment (Invitrogen), RNA was reverse transcribed into cDNA as previously described [33]. QPCR reactions were carried out as previously described [34], using the following primers:

- AKTIP Forward 5’-TCCACGCTTGGTGTTCGAT-3’;
- AKTIP Reverse 5’-TCACCTGAGGTGGGATCAACT-3’;
- lamin A/C Forward 5’-TGGAGGAGGTGGATGAGGAG-3’;
- lamin A/C Reverse 5’-CATTCTGGCGCTTGATCTGC-3’;
- lamin A Forward 5’-CTCCTACCTCCTGGGCAACT-3’;
- lamin A Forward 5’- AGGTCCCAGATTACATGATGCT-3’;
- lamin C Forward 5’- CTCAGTGACTGTGGTTGAGGA-3’;
- lamin C Reverse 5’- AGTGCAGGCTCGGCCTC-3’;
- GAPDH Forward 5’-TGGGCTACACTGAGCACCAG-3’;
- GAPDH Reverse 5’-GGGTGTCGCTGTTGAAGTCA-3’;
- and analyzed with the 2–ΔΔCq method as previously described [35].

For Western blotting, protein extracts were obtained as previously described [1] and quantified by Bradford assay. 100 µg of protein extracts were loaded onto pre-cast 4–12% gradient acrylamide gels (Novex, Life Technology). After electro-blotting, filters were incubated with anti-AKTIP (HPA041794 Sigma), anti-lamin A/C (sc-7292, Santa Cruz Biotechnology), anti laminB1 (sc-6017 Santa Cruz Biotechnology) and anti-actin-HRP conjugated (sc-1615, Santa Cruz Biotechnology) antibodies. Filters were then incubated with appropriate HRP-conjugated secondary antibody (sc-2004, sc-2005 Santa Cruz Biotechnology). Detection was performed using the enhanced chemiluminescence system (Clarity ECL, Biorad).

### Immunofluorescence and microscopy

Cells were seeded onto glass coverslips in 6-well plates, were pre-permeabilized according to [36] and fixed with 3.7% formaldehyde in PBS for 10 min. Cells were then permeabilized with 0.25% Triton X-100 in PBS for 5 min and treated with PBS 1%BSA for 30 min, and stained with primary antibodies in PBS 1% BSA for 1 h at RT. The following primary antibodies were used: anti-AKTIP (WH0064400M2 clone 2A11, Sigma), anti-lamin A/C (sc-6215, Santa Cruz Biotechnology), anti-lamin B1 [37], anti-TPR (ab84516, Abcam), anti-progerin (clone 13A4, Abcam), anti-Flag (clone M2, sc-F3165, Sigma Aldrich). Alexa488, Alexa568, Alexa647 or FITC conjugated secondary antibodies were applied in PBS for 45 min at RT. Nuclei were visualized using DAPI (4,6 diamidino-2-phenylindole) and coverslips were mounted in Vectashield H-1000.The slides were then examined with a Zeiss Axioplan epifluorescence microscope equipped with a CCD camera (CoolSnap HQ; Photometrics,). Grayscale images were pseudocolored and combined in Adobe Photoshop CC to create merged images.

For 3D-SIM imaging cells were seeded onto glass coverslips (high performance coverslips #1.5H Cat. #0,107,052—Marienfeld superior) in 6-well plates, were pre-permeabilized according to [36] and fixed with 3.7% formaldehyde in PBS for 10 min at RT and then incubated in 50 mM NH4Cl/PBS (15 min). Primary and secondary antibodies were applied in PBS BSA1% for 1 h at RT and washed in PBS. Acquisition was performed using a DeltaVision OMX v4 Blaze microscope (GE Healthcare, Singapore) with the BGR-FR filter drawer for acquisition of 3D-SIM images. An Olympus Plan Apochromat 100 × /1.4 PSF oil immersion objective lens was used with liquid-cooled Photometrics Evolve EM-CCD cameras for each channel. Fifteen images per section per channel were acquired with a z-spacing of 0.125 μm [38, 39]. Structured illumination reconstruction and wavelength alignment was done using the SoftWorX software (GE Healthcare). For A549 and MCF7 SIM imaging was performed using a Nikon Eclipse Ti equipped with: X-Light V3 spinning disk combined with a VCS (Video Confocal Super resolution) module (CrestOptics) based on structured illumination. LDI laser source (89 North) and Prime BSI Scientific CMOS (sCMOS) camera with 6.5 µm pixels (Photometrics) were used. The images were acquired by using Metamorph software version 7.10.2. (Molecular Devices) with a Nikon 100x/1.45 Plan Apochromat Lambda oil immersion objective at a z-spacing of 0.2 μm. In order to achieve super-resolution, raw data obtained by the VCS module were processed with a modified version of the joint Richardson-Lucy (jRL) algorithm [40–43], where the out of focus contribution of the signal has been explicitly added in the image formation model used in the jRL algorithm, and evaluated as a pixel-wise linear “scaled subtraction” [44] of the raw signal.

### Image analysis and quantification

Image analyses were performed using Imaris Software (Bitplane, Zurich, Switzerland) for 3D-SIM microscopy or NIS-Elements Software (Nikon) for A549 and MCF7 SIM images and using Image J/Fiji (National Institutes of Health, Bethesda, MD [45]) for the other images. For 3D-SIM images, co-localization between AKTIP and the indicated proteins was quantified as Pearson correlation coefficient, using the co-localization module of Imaris Software or the co-localization module of NIS-Element software. AKTIP spot counts were made using the spots module of Imaris Software. 3D volume reconstructions and movies generation from 3D-SIM data were done using Imaris Software.

AKTIP foci were identified in immunofluorescence images and analyzed using Analyze particles module of Image J on processed images through FFT bandpass filter (used set up: filter large structure down to 4pixels; filter small structures up to 4pixels; suppress stripes None; tolerance of direction 5%) and after watershed process to binary threshold (moments) images. To identify AKTIP foci at the rim, the processed pictures were superimposed on the corresponding DAPI images. Morphometric nuclei analyses were conducted on DAPI images using Analyze particles module of Images J and measuring shape descriptors parameters, circularity, roundness and solidity. Results are shown as mean ± SEM. Statistical analyses were conducted using unpaired two-tailed Student’s t-test using Prism software (Graphpad). *p*-values below 0.05 were considered significant and reported on graph as * *p* < 0.05; ** *p* < 0.01; *** *p* < 0.001. *P*-values > 0.05 were considered not significant and were not reported on graphs.

## Results

### AKTIP co-localization with lamins in HeLa cells

**Fig. 1:**
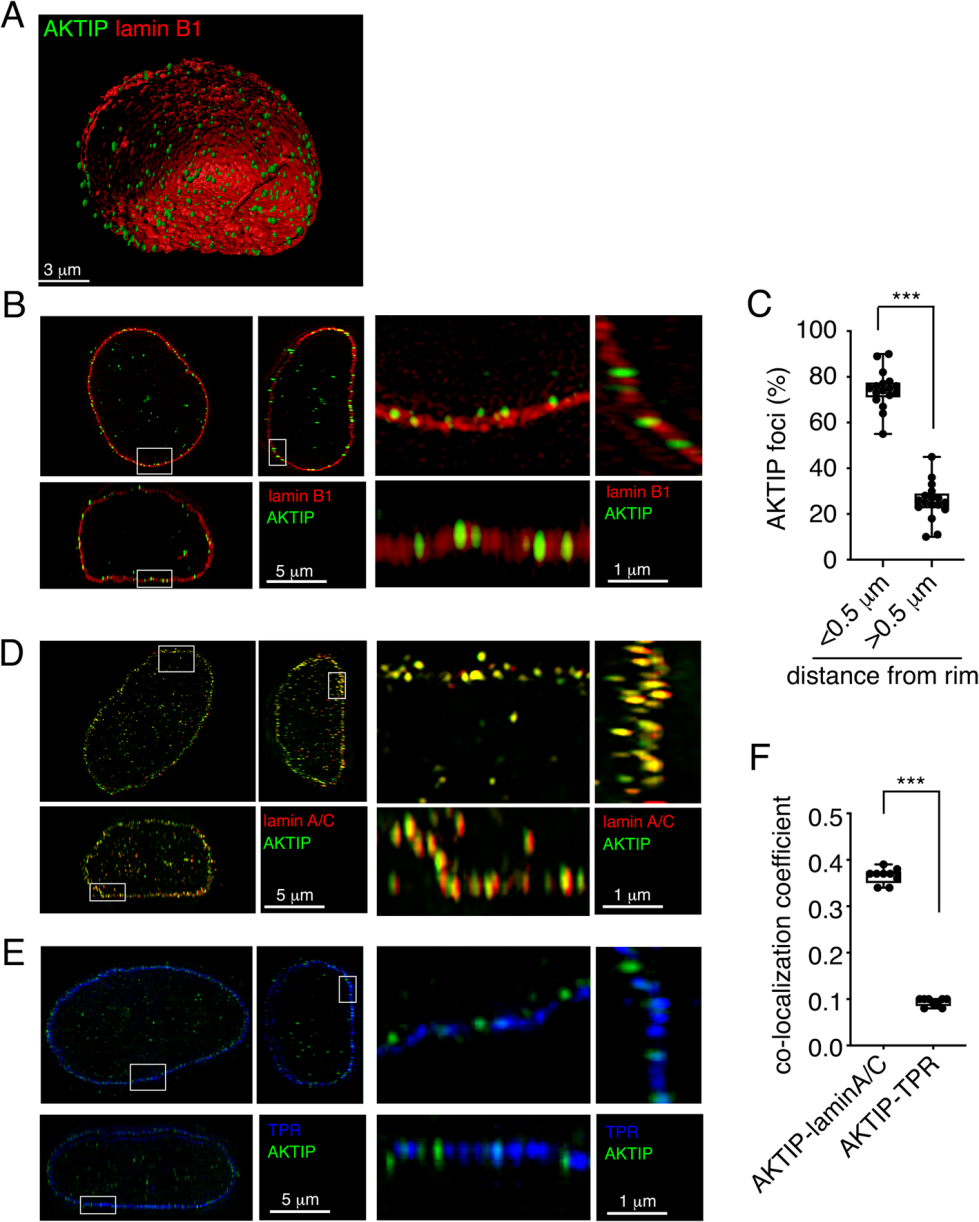
3D SIM imaging of the localization of AKTIP in HeLa cells (**A**, **B**) 3D rendering (**A**) and projections of an extended section viewed from orthogonal planes and magnified sections (**B**) from super resolution images of a HeLa nucleus labelled with anti-lamin B1 and anti-AKTIP antibodies. **C** Quantification of the percentage of AKTIP foci at a distance below or above 0.5 µm from the lamin B1 signal. **D** Projection of an extended section of a nucleus labelled with anti-lamin A/C and anti-AKTIP antibodies and magnified sections. **E** Projections of an extended section viewed from orthogonal planes of a nucleus labelled with anti-TPR and anti-AKTIP antibodies and magnified sections (**F**) Co-localization between AKTIP and the indicated proteins from (**D**) and (**E**) quantified as Pearson correlation coefficient. Each dot corresponds to a nucleus. Mean ± SEM are shown, images from 9 nuclei per cell condition were analyzed. ***p < 0.001 in unpaired Student t-test

As a first step to analyze AKTIP localization, we imaged with high resolution HeLa cancer cells. We used 3D structured illumination microscopy (3D SIM), which delivers images with nanometer scale resolution [46]. Lamin B1 staining was employed to visualize the nuclei (Fig. 1A). Co-labelling of HeLa cells with anti-lamin B1 and anti-AKTIP antibodies shows that AKTIP prevalently localizes as discrete foci enriched at a distance < 0.5 µm from the rim (Fig. 1A-C and Supplementary movie 1). Co-labelling of AKTIP and lamin A/C shows that AKTIP and lamin A/C signals co-localize (Fig. 1D, F and Supplementary movie 2). We next determined by semi-automatic analysis the relative percentage of AKTIP foci co-localizing with lamin A/C in the nucleoplasm and at the nuclear rim. This quantitative analysis showed that AKTIP co-localizes with lamin A/C more at the nuclear rim than at the nucleoplasm (Figure S1A). We next analyzed whether AKTIP recruitment at the nuclear rim depends on the level of lamin A. To address this point, we overexpressed lamin A in 293 T cells containing GFP-tagged AKTIP. We observed that, as compared to control GFP-AKTIP 293 T cells, lamin A overexpressing AKTIP-GFP cells show higher AKTIP staining at nuclear rim (Figure S1B). These data indicate that increased lamin A drives AKTIP at the nuclear rim.

Since a regular rim pearl pattern is typically observed for nuclear pore complexes (NPCs) [47], we next investigated the position of AKTIP foci relative to NPCs. We performed co-immunofluorescence using an anti-AKTIP antibody and an antibody directed towards the NPC basket protein TPR [47]. Images show the distribution at the rim of both TPR and AKTIP, with foci of apparent similar size but with distinct positions (Fig. 1E). This independent localization of TPR and AKTIP is quantitatively defined by a correlation coefficient < 0.1, in contrast to the robust correlation coefficient established for the signals of AKTIP and lamin A/C (Fig. 1F).

These data together show that in HeLa cells AKTIP is in proximity of lamin B1 and co-localizes with lamin A/C, while it has a distinct pattern at the rim with respect to the nucleoporin TPR.

### Comparative AKTIP localization in tumor cells

As a second step, we comparatively analyzed AKTIP in HeLa, MCF7 and A549. These three cell lines derive from different cancer types, namely, HeLa from cervical cancer, A549 from lung adenocarcinoma, and MCF7 from breast cancer (see Materials and Methods). We selected these cell lines because they express wild type p53, though at different levels (HeLa < A549 < MCF7) [48], while lamin A is expressed both in HeLa and A549 [49–51], but it is lowered in MCF7 [17, 52].

**Fig. 2:**
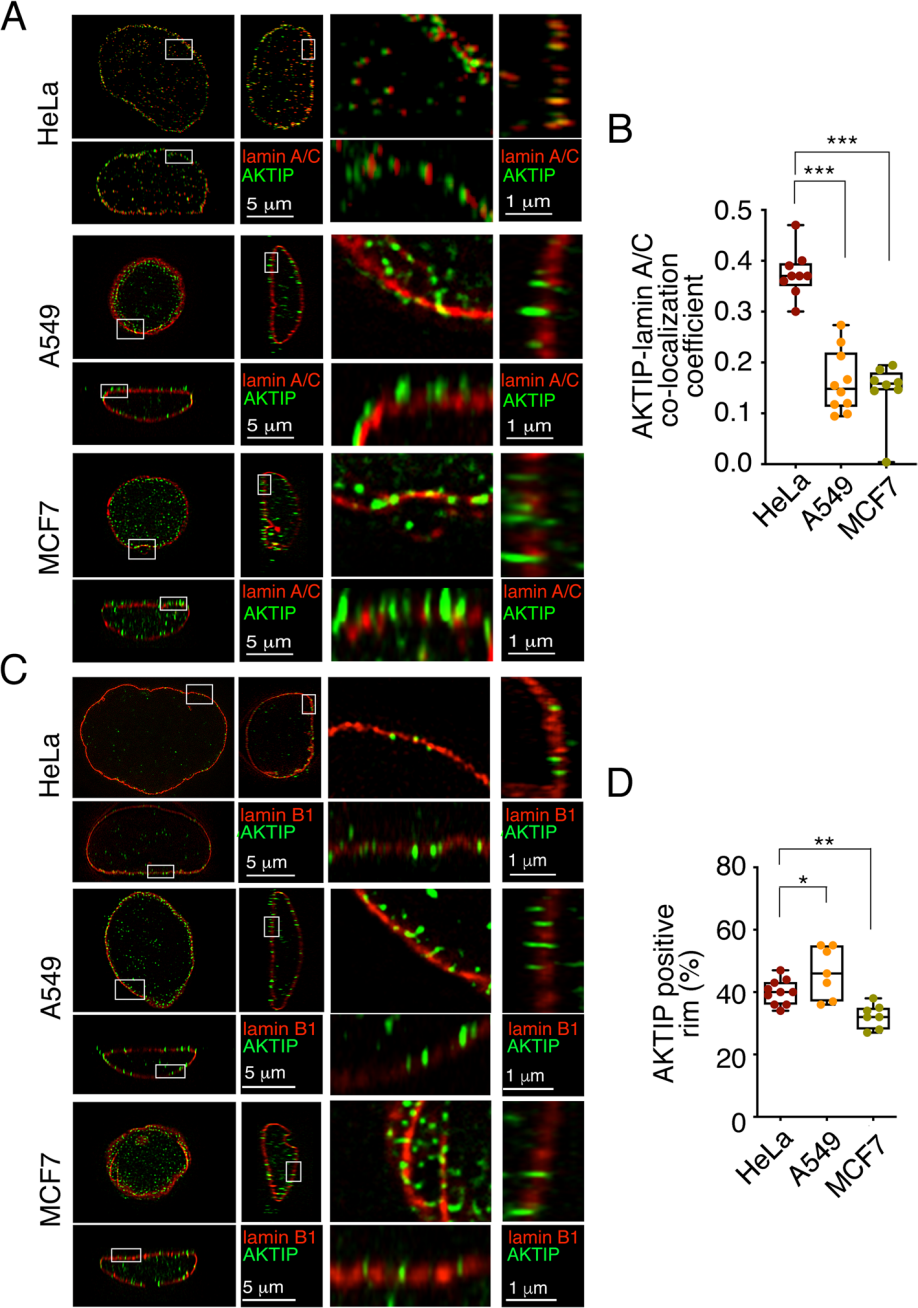
Comparative 3D SIM imaging of the localization of AKTIP in tumor cells (**A**) Projections of an extended section of Hela, A549 and MCF7 nuclei labelled with anti-lamin A/C and anti-AKTIP antibodies, viewed from orthogonal planes and magnified sections. **B** Pearson correlation coefficient of the co-localization between AKTIP and lamin A/C in A549 and MCF7 and HeLa cells. **C** Projections of an extended section of Hela, A549 and MCF7 nuclei labelled with anti-lamin B1 and anti-AKTIP antibodies, viewed from orthogonal planes and magnified sections. **D** Quantification of AKTIP foci distribution in HeLa, A549 and MCF7 cells measured as the percentage of AKTIP positive rim. In **B** and **D** each dot corresponds to a nucleus. A minimum of 7 nuclei were counted for each cell line. Mean ± SEM are shown. * *p* < 0.05, ** *p* < 0.01 in unpaired Student t-test, ***p < 0.001 in unpaired Student t-test

We analyzed the localization of AKTIP in parallel in the three cell types co-stained for lamin A/C and AKTIP (Fig. 2A, B). A549 cells show enrichment of AKTIP at the nuclear rim, however, AKTIP foci appear more spaced apart and show a reduced overlapping with the signal of lamin A/C, when compared to HeLa. MCF7 show high heterogeneity in lamin A/C fluorescence levels in the cell population. Additionally, anti-lamin A/C staining highlights the presence of nuclear wrinkles and blebs. AKTIP foci, despite being enriched at the nuclear rim, are spaced and only partially overlap with lamin A/C (Fig. 2A). These observations are confirmed by quantitative analysis. We observe a significant reduction of the correlation index of the signals from AKTIP and lamin A/C in A549 and MCF7, as compared to HeLa cells. These data point to a reduced association between AKTIP and lamin A/C in the nucleus, both in the nucleoplasm and at the nuclear rim in A549 and MCF7 (Fig. 2B). We next analyzed cells co-stained for AKTIP and lamin B1. This analysis confirms that AKTIP is present at the rim in the three cell types, but also highlights a specific pattern in MCF7 (Fig. 2C). Namely, in MCF7, lamin B1 staining highlights multiple nuclear defects, wrinkles, blebs, and rim interruptions. Image quantification indicates that the portion of nuclear rim occupied by AKTIP is significantly reduced in MCF7 as compared to HeLa and to A549. These data suggest that the localization of AKTIP at the nuclear rim is slightly increased in A549 and reduced in MCF7, as compared to HeLa cells (Fig. 2D). These findings are also consistent with the observation that increased lamin A promotes the localization of AKTIP localization at the nuclear rim (Figure S1B).

These results indicate heterogeneous lamin expression and nuclear organization patterns in the three tumor cell types and highlight that in MCF7 breast tumor cells AKTIP is mislocalized.

### AKTIP localization in non-transformed cells with lamin alterations

With the goal of assessing whether AKTIP mislocalization was generated by lamin alterations, we used two cell models with known LMNA mutations: non-transformed fibroblasts derived from HGPS LMNA c.1824C > T p.Gly608Gly and from EDMD2 LMNA c.775 T > G patients. As control in this experimental setting, we used non-transformed wild type fibroblasts derived from a healthy donor.

**Fig. 3:**
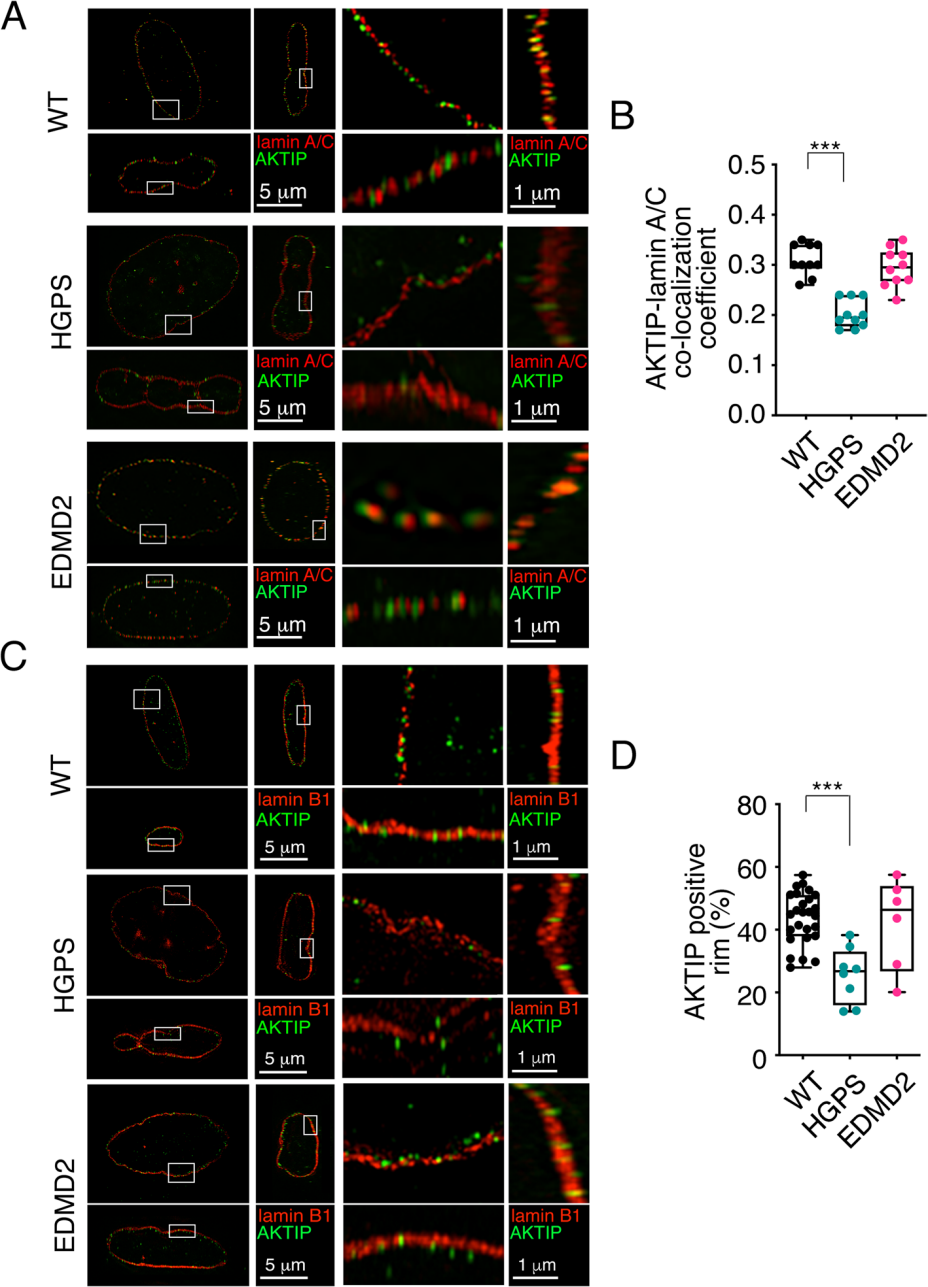
Comparative 3D SIM imaging of the localization of AKTIP in non-transformed laminopathic cells (**A**) Projections of an extended section of non-transformed wild type, HGPS and EDMD2 patient derived fibroblast nuclei labelled with anti-lamin A/C and anti-AKTIP antibodies, viewed from orthogonal planes and magnified sections. **B** Pearson correlation coefficient to measure the co-localization between AKTIP and lamin A/C in wild type, HGPS and EDMD2 cells. **C** Projections of an extended section of non-transformed wild type, HGPS and EDMD2 nuclei labelled with anti-lamin B1 and anti-AKTIP antibody, viewed from orthogonal planes and magnified sections. **D** Percentage of AKTIP positive rim in wild type, HGPS and EDMD2 cells. In **B** and **D** each dot corresponds to a nucleus. A minimum of 7 nuclei were counted for each cell type. Mean ± SEM are shown. ***p < 0.001 in unpaired Student t-test

Orthogonal planes of lamin A/C-AKTIP stained nuclei show the co-localization of AKTIP and lamin A/C in non-transformed wild type fibroblasts (Fig. 3A). In HGPS and EDMD2 cells the images and the quantitative analysis show a selective reduction of co-localization of AKTIP and lamin A/C in HGPS cells as compared to wild type and to EDMD2 (Fig. 3A, B). Orthogonal planes of lamin B1/AKTIP stained nuclei reveal a pattern similar to that observed in lamin A/C-stained nuclei (Fig. 3C). The localization of AKTIP at the rim is evident in wild type nuclei, significantly lost in HGPS cells, and modestly, but not significantly, altered in EDMD2 nuclei (Fig. 3C, D).

These results show that AKTIP is mislocalized in HGPS, but not in EDMD2 cells, although both cell types have LMNA mutation. Given the profoundly altered shape of HGPS nuclei, the data suggest that aberrant nuclear morphology could contribute to AKTIP mislocalization. Moreover, AKTIP mislocalization in HGPS cells indicates that AKTIP localization is independent of the p53 gene status, which is wild type in these cells.

### Quantitative analysis of lamin A/C expression and nuclear morphology

To explore the interrelation between AKTIP mislocalization, lamin expression, and nuclear morphology, we quantified these traits in all cell models, including tumor and non-transformed laminopathic cells.

**Fig. 4:**
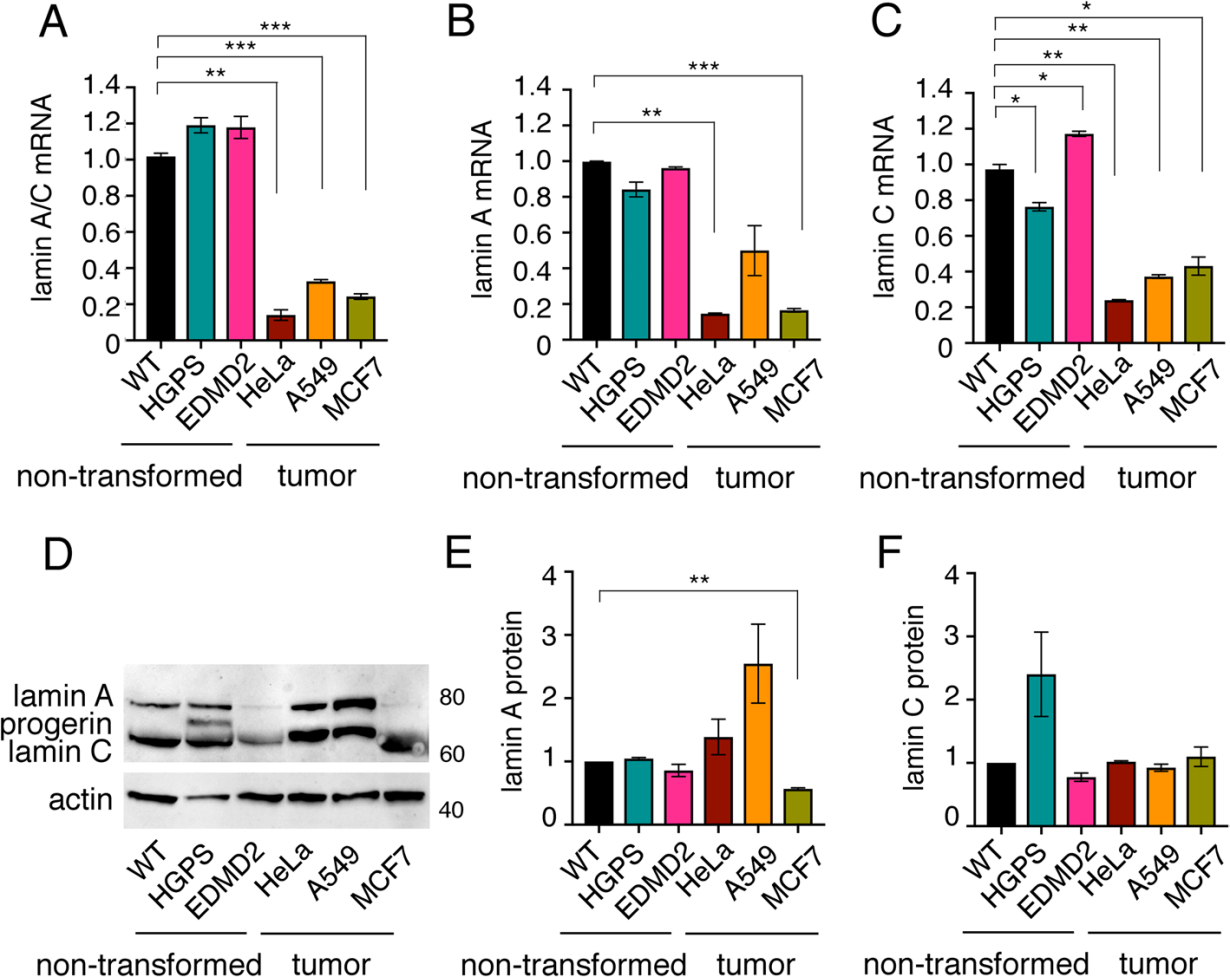
Lamin expression in tumor and non-transformed laminopathic cells (**A**) RT Q-PCR of LMNA mRNA. **B** RT Q-PCR of lamin A mRNA. **C** RT Q-PCR of lamin C mRNA. **D-F** Western Blotting and relative quantification showing lamin A, lamin C and progerin protein levels. Actin was used as loading control. In **A**-**C** and **E**–**F** for each sample, relative fold change respect to non-transformed wild type fibroblasts is shown. Mean values of two independent experiments ± SEM are shown. *p < 0.05, ** *p* < 0.01, ***p < 0.001, in unpaired Student t-test

We measured *LMNA* expression by RT-QPCR using oligos recognizing both lamin A and lamin C. This analysis shows that the levels of *LMNA* mRNA are significantly reduced in the three tumor cell lines (HeLa, A549, and MCF7), as compared to non-transformed cells (wild type, HGPS and EDMD2) (Fig. 4A). Primers targeting either lamin A or C, confirm these results (Fig. 4B, C). We next measured the level of lamins A and C by Western Blotting (Fig. 4D-F). We observe the reduction of lamin A in MCF7 (Fig. 4D, E) and the presence of the progerin band in HGPS cells (Fig. 4D). We next evaluated the levels of lamin B1 by Western Blotting (Figure S2A). We observe a reduction of lamin B1 in HGPS, EDMD2 cells and in A549 and MCF7 tumor cells, as compared to non-transformed wild type fibroblasts (Figure S2A, B).

**Fig. 5:**
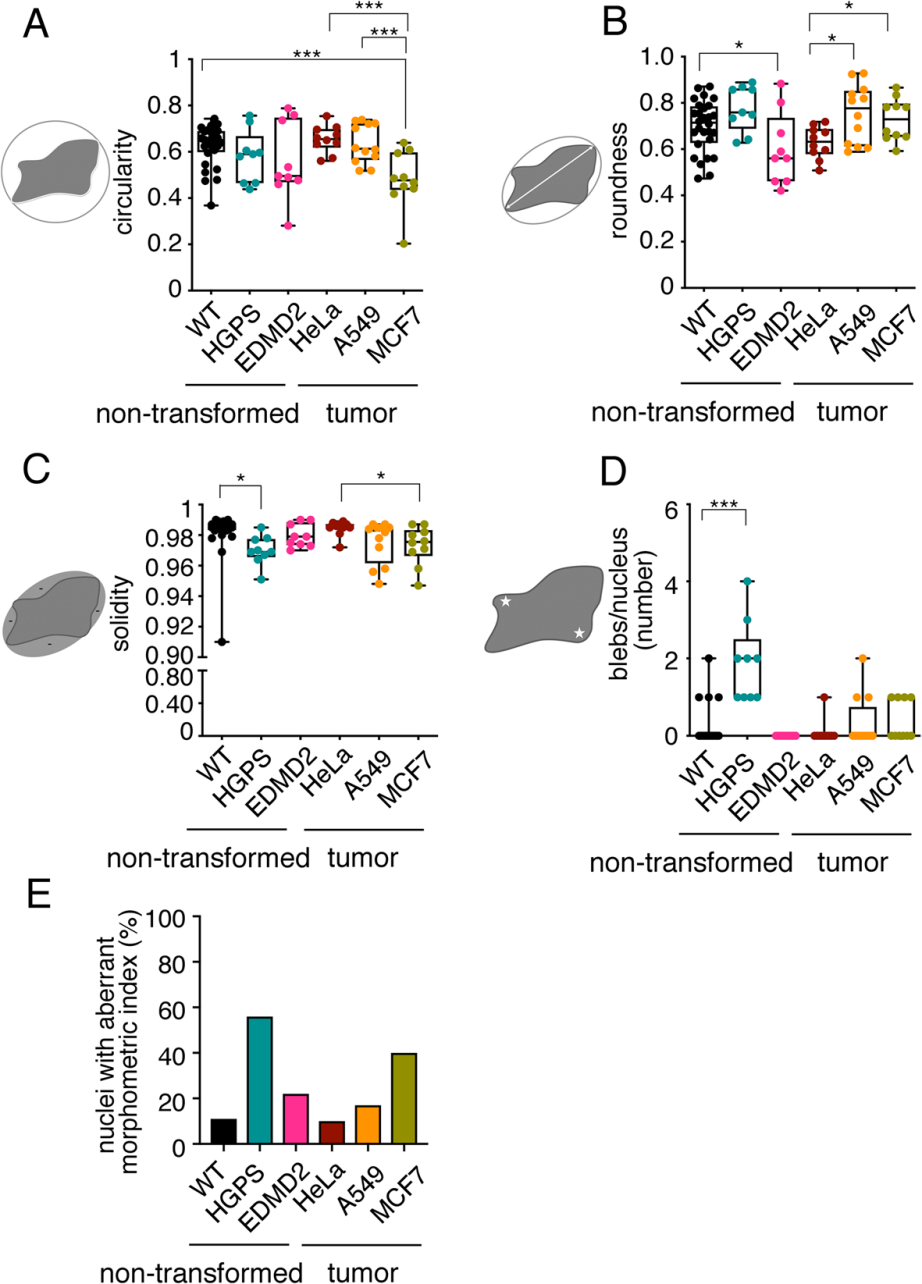
Morphometric analysis of tumor and non-transformed laminopathic cell nuclei (**A-D**) Quantification of morphometric parameters for nuclei of the indicated cell types (x axis). **E** Percentage of nuclei that simultaneously show lower circularity and solidity indices and a higher roundness index respect to the mean values of non-transformed wild type fibroblast. Each dot corresponds to a nucleus. A minimum of 7 nuclei were counted for each cell type Mean value and minimum and maximum values are shown. * *p* < 0.05, ** *p* < 0.01; ***p < 0.001 in unpaired Student t-test

We next performed morphometric measurements of the nuclei of the different cell types. By semi-automated quantification, we monitored the circularity, solidity, and roundness indices and the number of nuclear blebs (Fig. 5). This analysis shows that the circularity [4π(area/perimeter^2^)] is reduced in MCF7 (Fig. 5A). Roundness, defined by the Eq. (4xarea)/(πxmajor axis^2^), is decreased in EDMD2 (Fig. 5B). Solidity, calculated as the ratio of area of the nucleus and the area of its convex hull shape, is slightly but significantly reduced in HGPS and MCF7 (Fig. 5C). Blebs are abundant in HGPS (Fig. 5D). To condense these data into a single morphometric index defining nuclear aberration, we considered the percentage of nuclei that simultaneously showed lower circularity and solidity indices and higher roundness index. Based on this cumulative morphometric index, HGPS and MCF7 are the cells with the highest frequency of nuclear aberration, *i.e.*, 56% and 40%, respectively (Fig. 5E).

### Correlation between AKTIP localization, lamin A/C expression and nuclear morphology

**Fig. 6:**
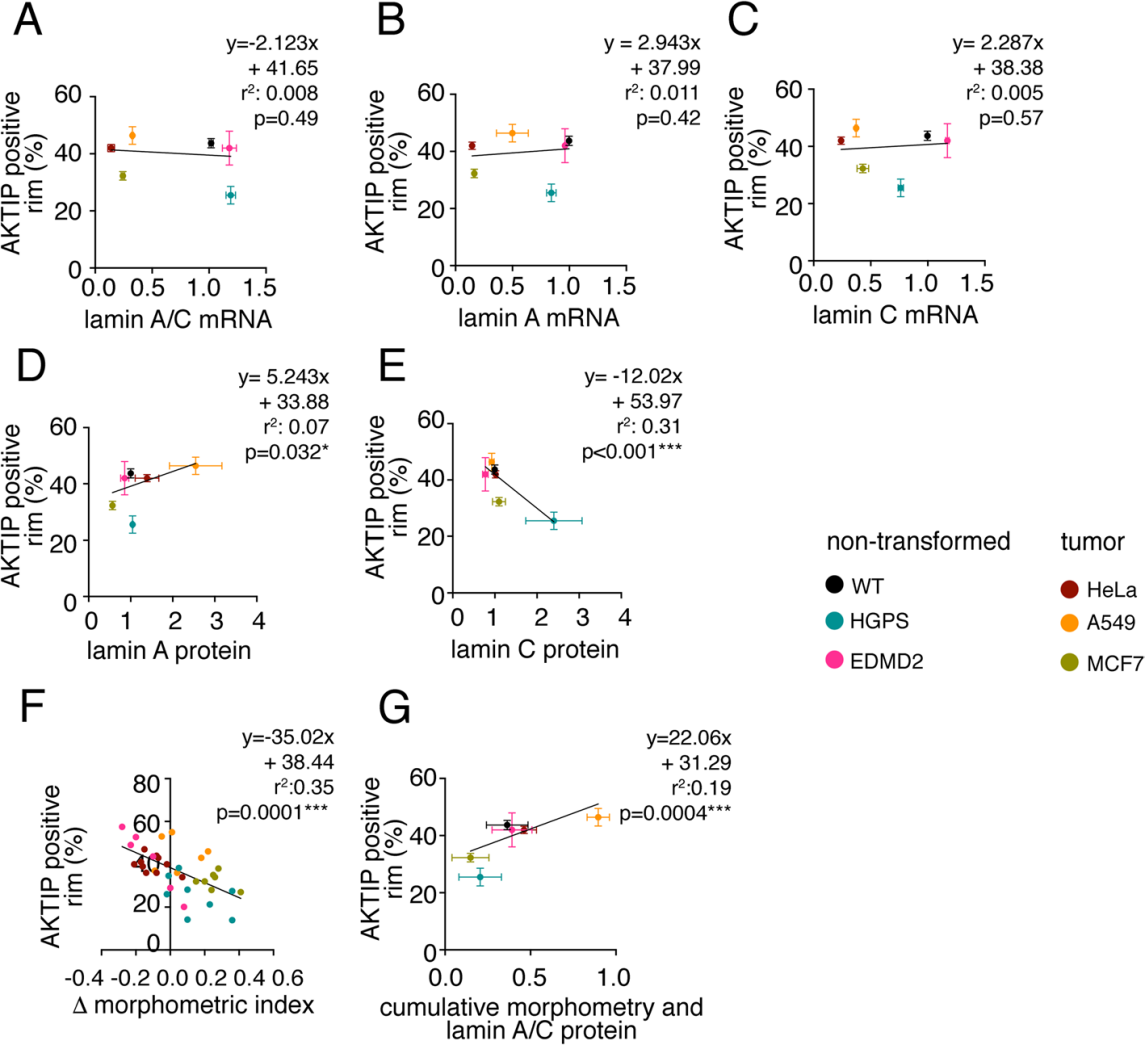
Correlation analysis of AKTIP localization with lamin expression and morphometric index. **A-C** AKTIP localization at the rim, expressed as the percentage of AKTIP positive rim, does not significantly correlate with lamin mRNA levels quantified by RT Q-PCR.** D**, **E** Correlation between percentage of AKTIP positive rim and lamin A and lamin C expression quantified by Western Blotting. **F** Inverse correlation between AKTIP localization at the rim and morphometric index, calculated for each nucleus as the sum of the differences between each morphometric index and their mean value in wild type non-transformed fibroblasts. **G** Correlation between AKTIP positioning at the rim and cumulative parameter obtained merging for each type the ratio between lamin A and lamin C proteins levels from WB quantification and morphometric index as in F proteins ratio and morphometric index. In **F** each dot corresponds to a nucleus. In **A**-**E**, and **G** mean values ± SEM are shown. Linear regression values are shown

We next wanted to establish to what extent AKTIP mislocalization correlated with lamin expression and nuclear morphology. To this goal, we calculated correlative values based on linear regression between the different parameters and report the *r* and *p* values performed on all cell types (Fig. 6). No significant correlation (*p* > 0.1) is observed between lamin mRNA levels and the positioning of AKTIP (Fig. 6A-C). When considering protein expression, we observed no significant correlation (*p* > 0.05) between lamin B1 levels and between the ratio lamin A/lamin B1 levels and AKTIP positioning at rim (Fig. 6D, E and Figure S2C-D). On the contrary, a negative correlation is present for AKTIP positioning at the rim and the level of lamin C (*r*^2^ = 0.31, *p* < 0.001). When we consider the association between the presence of AKTIP at the nuclear rim and the level of nuclear aberration, we observe a significant negative correlation (*r*^2^ = 0.35, *p* = 0.0001; Fig. 6F). Merging the lamin protein expression parameter with the cumulative morphometric index, we recapitulated the information on the different cell types. Namely, the mislocalization of AKTIP, evident in HGPS and MCF7 cells, is associated with alterations of the lamin protein and of nuclear morphology (Fig. 6G).

Considering the involvement of AKTIP in several essential cellular processes [1, 2, 6], we next evaluated whether there was a correlation between the localization of AKTIP at the nuclear rim and the proliferation rate of the tumor cell lines. To this aim, we monitored the proliferation rate of HeLa, A549 and MCF7 (Figure S2E, F). We observe that the proliferation rate of HeLa cells is higher than that of A549 and MCF7 (Figure S2E). We next calculated the correlative values based on linear regression between population doubling and AKTIP positioning at the rim. We did not find a correlation, which suggests that the two aspects are not interdependent (Figure S2F).

### AKTIP localization in HeLa cells expressing HGPS mutant lamin

**Fig. 7:**
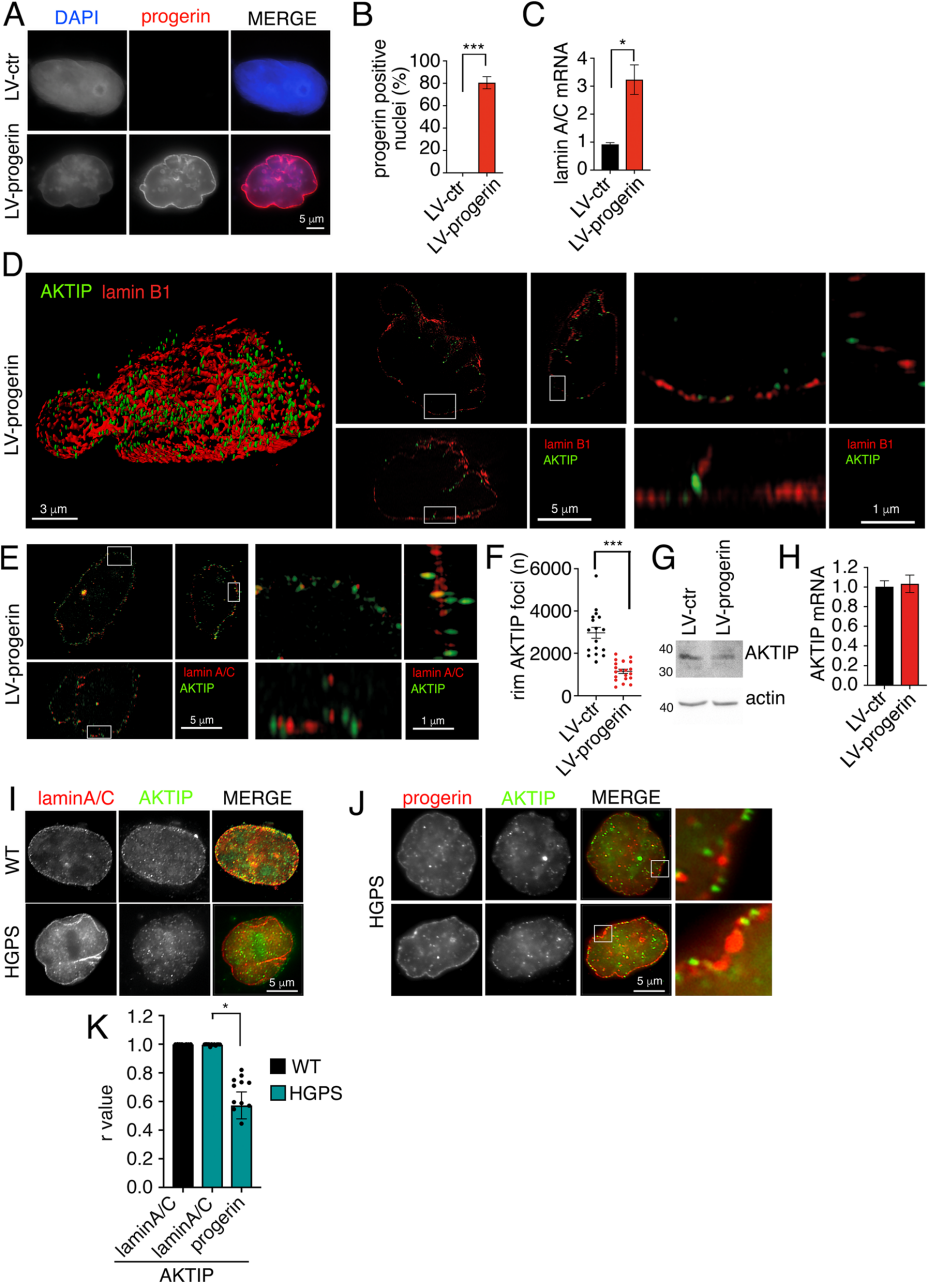
AKTIP localization in progerin expressing HeLa cells (**A**, **B**) Images (**A**) and quantification (**B**) of DAPI and progerin stained nuclei of HeLa cells transduced with a control vector (LV-ctr) or with progerin expressing vector (LV-progerin) and stained for progerin and DAPI. **C** RT Q-PCR of LMNA mRNA (including progerin) expression. In **B**-**C** mean values of two independent experiments ± SEM are shown. In **A** 100 nuclei were counted for each condition **D** 3D rendering, projections of an extended section viewed from orthogonal planes and magnified sections of LV-progerin HeLa nucleus labelled with anti-lamin B1 and anti-AKTIP antibodies. **E** Projections of an extended section viewed from orthogonal planes of images of LV-progerin HeLa nuclei stained with anti-lamin A and anti-AKTIP antibodies, and magnified sections. **F** Quantification of AKTIP foci at a distance > 0.5 µm from lamin B1 in control (LV-ctr) and progerin (LV-progerin,) HeLa cells. Each dot corresponds to a nucleus. A minimum of 15 nuclei were analyzed for each condition. **G** Western blotting using anti-AKTIP antibody in control (LV-ctr) and progerin expressing (LV-progerin) HeLa cells. Actin was used as loading control. **H** RT Q-PCR of AKTIP mRNA in control (LV-ctr) and progerin expressing (LV-progerin) HeLa cells. Mean values of two independent experiments ± SEM are shown. **I** Images of wild type and HGPS fibroblasts stained for lamin A/C and AKTIP showing in merge that there is no AKTIP accumulation at the site in which lamin A/C is accumulated due to progerin expression. **J** Images of HGPS fibroblasts stained for progerin and AKTIP and relative enlargements showing that there is no AKTIP signal at the site of progerin accumulation at nuclear rim. DNA was stained with DAPI. **K** Pearson correlation value of intensity plot of AKTIP-lamin A/C distribution at nuclear rim in wild type fibroblasts, and AKTIP-lamin A/C and of AKTIP-progerin distribution at nuclear rim in HGPS fibroblasts. Each dot corresponds to a nucleus. A minimum of 10 nuclei were evaluated for each condition. Mean values ± SEM are shown. *p < 0.05, ***p < 0.001, in unpaired Student t-test

As described in the previous paragraph, the integrated analysis of lamin expression, nuclear morphology and AKTIP distribution shows that the positioning of AKTIP is influenced not only by lamin expression, but also by nuclear morphology. To validate this concept, we generated HeLa cells expressing the HGPS mutant lamin progerin. These cells were obtained using a lentiviral vector encoding progerin (LV-progerin). We firstly monitored the expression of progerin by immunofluorescence using an anti-progerin antibody (Fig. 7A, B) and by RT-QPCR (Fig. 7C). We next performed reconstruction of nuclei stained with anti-lamin B1 antibody (Fig. 7D). AKTIP is mislocalized in LV-progerin HeLa cells, indeed we observe a reduced number of AKTIP foci at nuclear rim that appear more spaced apart as compared to HeLa transduced with a control vector (LV-ctr). We successively performed the analysis of AKTIP on LV-progerin HeLa in nuclei stained with anti-lamin A/C antibody (Fig. 7E). DAPI and anti-AKTIP antibody staining show that LV-progerin determines a reduction in the number of AKTIP foci at the rim as compared to LV-ctr HeLa (from 2966/cell in LV-ctr to 1157/cell in LV-progerin; Fig. 7F). To exclude that the reduction of AKTIP foci was due to decreased AKTIP expression, we monitored AKTIP protein and mRNA levels by Western blotting and RT-QPCR, respectively. We observed no difference in the expression of AKTIP in LV-progerin HeLa cells compared to LV-ctr (Fig. 7G, H). We next wanted to exclude the possibility that the aberrant AKTIP localization observed in the presence of progerin was due to physical sequestration of AKTIP by progerin. Indeed, it was previously demonstrated that progerin sequesters some of lamin A interactors. This has been suggested to contribute to the HGPS pathological phenotype [53–56]. To investigate if AKTIP was sequestered by progerin, we used HPGS cells. Co-staining of the cells for lamin A/C, AKTIP, and progerin shows the profile of these proteins at the nuclear rim (Fig. 7I-K). The quantification of the signals shows that AKTIP has a similar distribution profile at the nuclear rim to that of lamin A/C in both control and HGPS cells (Fig. 7K). AKTIP and progerin, on the other hand, show different distribution profiles (Fig. 7K). This aspect is also highlighted by the absence of AKTIP signal in progerin aggregates (Fig. 7J), indicating that AKTIP reduction at the rim is not due to sequestration by progerin.

Taken together these experiments show that when aberrant HGPS lamin is expressed in tumor cells it can induce alteration of nuclear morphology and a robust mislocalization of AKTIP.

## Discussion

Lamins are key nuclear components, which control many cell functions and shape nuclear morphology. Lamin alterations and nuclear morphology have long been studied in cancer [13–15]. Lamin expression is altered in many tumors and cancer cell lines, but the impact of this alteration on the disease is still confounding. For instance, both reduced and increased levels of lamin A/C have been associated with poor cancer prognosis [17–19, 21, 22]. In more general terms, the data on the link between lamin and cancer suggest that the mechanistic implication of lamin is multifaceted and, on the other hand, that the measure of its expression is not sufficient to predict its impact on the disease.

Concerning the relation of nuclear morphology with cancer, the clinical data indicate that nuclear morphometrics is a relatively accurate approach for the diagnosis of late-stage cancer [57]. However, the use of nuclear morphometrics for early diagnosis is still a challenge. To overcome this difficulty, the forefront of this area of study proposes the usage of nuclear morphometrics at a single-cell resolution level, combined with artificial intelligence analyses of the images [57]. This approach can be improved even more by applying micromechanical manipulations to the test samples, to mimic the mechanical pressure of tumor microenvironments [58, 59]. In this experimental setting, the sample response to perturbations depends on the properties of the cells, thus it can be used as a source for further dissection, measurement, and prediction of the cancer phenotype. These approaches have, however, limitations. These are especially critical when considering the switch from bench to bedside. Indeed, a single and integrated analysis based on single-cell resolution, artificial intelligence, and mechanical manipulation, requires the suitability of the cancer clinical specimens and highly competitive dedicated facilities.

Another way for the use of nuclear morphology and lamin expression for cancer prognosis is to perform a refined integrative analysis of these two parameters. This approach is being implemented in laminopathies; rare genetic conditions due to genetic mutations in the lamin encoding gene *LMNA*. The involvement of different tissues and the different clinical symptoms in laminopathies suggest that the pathomolecular events downstream to LMNA mutations are complex and heterogeneous. For example, patients with HGPS suffer from premature aging, growth retardation, loss of subcutaneous fat, reduction in bone density, poor muscle development, and succumb to the disease at an early age [60, 61]. In contrast, laminopathic EDMD mutations cause contractures, muscle wasting, and cardiomyopathy [62]. To respond to the complexity of this genotype-to-phenotype relationship, to reconstruct and interpret the pathomolecular cascade occurring in laminopathic patients, recent works are studying not only the mutation of lamins per se, but also how lamin mutations control the morphology of the nucleus and, consequently, intranuclear elements [63–67]. Such an approach could be applied to cancer. This way of analyzing the problem is expected to be helpful in disease prognosis. In addition, it provides hints on the molecular mechanistic elements that are conditioned by lamin and nuclear alterations in cancer. This could be especially relevant in the study of tumor-associated factors residing at the nuclear rim.

AKTIP is a protein enriched at the nuclear rim [6] associated with cancer [2]. It is essential for cell survival, it is required to ensure the integrity of telomeres and genome [1], and, in vivo, the depletion of the mouse counterpart of AKTIP contributes to cancer aggressiveness [2]. AKTIP shares similarity with the tumor-associated factor TSG101. We discovered that AKTIP can genetically and physically interact with TSG101 [4]. In this study, we analyzed the positioning of AKTIP with super resolution and asked whether the distribution of AKTIP was altered in cancer cells and whether this was associated with alterations of lamins and/or with nuclear morphology.

We report that AKTIP is in close proximity with lamin B1, but specifically co-localizes with lamin A. This is reinforced by the observation that lamin A/C overexpression impacts on AKTIP recruitment at the rim. This association suggests a putative functional link of AKTIP with lamin A. This aspect could be of help to mechanistically interpret the partial phenotype overlap between progeroid mice and mice depleted of Ft1, the mouse orthologue of AKTIP. It could be speculated that this overlap is due to a functional association between AKTIP and lamin A [1, 2, 6].

NPCs are intertwined with the organization of the lamina [68, 69]. For example, cells depleted for lamin B or for lamin A/C display clustering of NPCs. In HGPS cells it has been hypothesized that the alteration of the nuclear shape drives NPC clustering [70, 71]. Despite the similarity between the pearl pattern of AKTIP at the rim and the distribution of NPCs, we found a distinct positioning of AKTIP and the NPC component TPR [46]. Since we previously demonstrated that the reduction of AKTIP expression triggers nuclear misshaping [6], in future work, it will be interesting to analyze if TPR alterations are driven by AKTIP depletion as in HGPS cells.

We next focused on cancer cells MCF7, HeLa and A549. We selected these three types because of their different origin and of their extensive previous characterization, including their lamin and p53 status [6, 17, 48–52]. We herein report that AKTIP positioning at the rim is altered in the breast cancer cell line MCF7 and not in HeLa and A549. Since p53 is wild type in our MCF7 cells [48], the finding that AKTIP is mislocalized these cells indicates that the p53 status does not have a role in determining AKTIP localization. However, at this point, we cannot rule out the possibility that gain-of-function mutations of p53 might alter this localization. We also show that AKTIP alteration is observable in a HeLa-progerin model system, in which we combined the presence of a defined lamin mutation with a cancer cell setting. These data suggest that AKTIP can be mislocalized in cancer cells with lamin alterations and this could have an impact on the disease phenotype. We also report that it is the combined alteration of lamin and nuclear morphology that influences the localization of the tumor-associated factor AKTIP. The fact that our results highlight that lamin alterations per se are not predictive of AKTIP mislocalization has multiple conceptual spin offs. The data suggest that for predicting a potential implication of AKTIP in cancer cells, lamin alterations should be monitored in parallel with nuclear morphology. Secondly, the data embrace the idea that the usage of lamin expression as potential prognostic biomarker in cancer is reinforced by a combined analysis of other biomarkers.

AKTIP is implicated in essential cellular processes including telomere metabolism and cell division [1, 2, 6], and it is possible that the mislocalization of AKTIP in MCF7 alters its function possibly contributing to the tumor phenotype. It is interesting to note in relation with this aspect that Cbioportal (www.cbioportal.org) and Oncomine [72] data sets report that AKTIP expression is altered in tumor. Additionally, the Human cell atlas (www.proteinatlas.org) indicates AKTIP as a prognostic marker of survival in renal cancers. This information taken together, suggest that AKTIP delocalization linked to lamin alterations could be further investigated as a mechanistic path to cancer disease.

This study also opens new insights for laminopathies. Indeed, consistently with the close association of AKTIP with the nuclear rim and with lamin A, the analysis of nuclei with either exogenous expression of progerin, or with the endogenous HGPS mutation, reveals that progerin profoundly impinges on AKTIP distribution, which suggests that AKTIP dysfunction could be a co-element in the progeroid disease. On the other hand, we do not see significant alterations of AKTIP in EDMD2 cells. Our data also highlight that the mislocalization of AKTIP in HGPS cells is not related to progerin sequestration, but rather to the specific morphological alterations of HGPS nuclei, *i.e*., their altered solidity and circularity. It is to note that HGPS cell abnormalities have also been associated to permanent protein farnesylation [73, 74]. We cannot exclude at this stage that this aspect could have an influence on AKTIP localization.

## Conclusion

In conclusion, this work, on one hand, contributes to defining the relationship between AKTIP, lamin, and nuclear alterations in cancer cells. On the other hand, it points to AKTIP dissociation from the nuclear rim as being one of the consequences of nuclear morphological changes caused by the HGPS LMNA mutation. Given that AKTIP is required for correct telomere function and genome integrity [1], its aberrant distribution in both cancer and laminopathic cells could be considered not only as a potential tool in disease prognosis, but also as a putative co-driver of the disease phenotypes. Overall, these results pave the way of next translational evaluation to validate the use of this combined analytical approach as risk biomarker.

## Supplementary Information

**Additional file 1: Supplementary movie 1.** Reconstruction of AKTIP localization in the nucleus labelled for lamin B1. 3D volume rendering of an HeLa nucleus stained with anti-lamin B1 (red) and AKTIP (green) and imaged with 3D-SIM. The volume rendering and the movie generation were performed with IMARIS software (Bitplane, Oxford Instruments).

**Additional file 2: Supplementary movie 2.** Reconstruction of AKTIP localization in the nucleus labelled for lamin A/C. 3D volume rendering of an HeLa nucleus stained with anti-lamin A/C (red) and AKTIP (green) and imaged with 3D-SIM. In gray the AKTIP-lamin A/C co-localization surfaces are shown. The volume rendering and the movie generation were performed with IMARIS software (Bitplane, Oxford Instruments).

**Additional file 3: Supplementary Figure S1.** Distribution of AKTIP-lamin A/C co-localizing foci and AKTIP localization in lamin A overexpressing cells. (A) Percentage of AKTIP foci that co-localizes with lamin A/C signal at rim and at nucleoplasm calculated on the total of AKTIP foci at rim and nucleoplasm respectively in HeLa cells. Each dot represents a nucleus. Images from 10 HeLa nuclei were analyzed. Mean values ± SEM are shown. *** *p*<0.01, in unpaired Student t-test. (B) Representative images and magnification from control and Flag-lamin A transfected AKTIP-GFP (green) 293T cells stained with anti-Flag antibody (red). DNA was stained with DAPI.

**Additional file 4: Figure S2.** Correlation analysis of AKTIP localization with lamin B1 expression and with proliferation rate of tumor cells. (A-B) Western Blotting and relative quantification showing lamin B1 levels. Actin was used as loading control. In (B) for each sample, relative fold change with respect to non-transformed wild type fibroblasts is shown. Mean values of two independent experiments ± SEM are shown. *p<0.05, ** *p*<0.01, in unpaired Student t-test. (C-D) Correlation between the percentage of AKTIP positive rim and lamin B1 (C), and between the ratio between lamin A (from Figure 4 D-E) and lamin B1 protein levels (D) quantified by Western Blotting. Mean values ± SEM are shown. Linear regression values are shown. (E) Cumulative population doubling of tumor cells. Mean values of three independent experiments ± SEM are shown (F) Correlation between the percentage of AKTIP positive rim and population doubling of HeLa, A549, MCF7 cells. Mean values ± SEM are shown. Linear regression values are shown.

## Data Availability

Data sharing is not applicable to this article as no datasets were generated or analyzed during the current study.

## Abbreviations

ESCRT: Endosomal Sorting Complex Required for Transport
EDMD: Emery-Dreifuss Muscular Dystrophy
HGPS: Hutchinson Gilford Progeria Syndrome
SIM: Structured illumination microscopy
NPCs: Nuclear pore complexes
LV: Lentivector
ctr: Control
SEM: Standard error mean
